# Supplementary material for: The Dynamics of Initiative in Communication Networks
Source: PLoS One. 2016 Apr 28;11(4):e0154442. doi: 10.1371/journal.pone.0154442 (PMC4849575; doi:10.1371/journal.pone.0154442)
Supplement: S1 Text — Short description of data used in the manuscript. (PDF) [file pone.0154442.s001.pdf]

This is a short note describing the data format. The data is saved in text format. Users are represented in vertical order followed by a whitespace. Each line is a relationship for the particular user. The line represents a series of initiatives, where 1 denotes an incoming initiative and 2 denotes an outgoing initiative.
